# Development and characterization of phantoms to investigate the Flash effect with *Drosophila melanogaster* at an ultra-high dose rate radiotherapy linac

**DOI:** 10.1016/j.phro.2025.100835

**Published:** 2025-09-15

**Authors:** Riccardo Dal Bello, Marvin Kreuzer, Irene Vetrugno, Jamie C. Little, Rafael Kranzer, Stefan Schischke, Lily Bossin, Eduardo Gardenali Yukihara, Matthias Guckenberger, Martin Pruschy, Stephanie Tanadini-Lang

**Affiliations:** a Department of Radiation Oncology, University Hospital Zurich and University of Zurich, Zurich, Switzerland; b Laboratory for Applied Radiobiology, Department of Radiation Oncology, University Hospital Zurich, University of Zurich, Zurich, Switzerland; c Department of Molecular Life Sciences, University of Zurich, Zurich, Switzerland; d PTW-Freiburg, Freiburg im Breisgau, Germany; e RadPro International GmbH, Wermelskirchen, Germany; f Department of Radiation Safety and Security, Paul Scherrer Institute, Villigen PSI, Switzerland

**Keywords:** Drosophila melanogaster, Flash, Ultra-high dose rate, Linac, Electron

## Abstract

**Background and purpose:**

Ultra-high dose rate (UHDR) radiotherapy may widen the therapeutic window thanks to the Flash effect. Experimental linear accelerators have been converted to UHDR to collect pre-clinical evidence. Increasing the accessibility, throughput and investigating additional biological endpoints is key for deciphering the mechanism of the Flash effect. The aim of this study was to develop and characterise an experimental platform for UHDR experiments with *Drosophila melanogaster*, i.e. the fruit fly.

**Materials and methods:**

A clinical linear accelerator was modified to deliver 16 MeV electron beams in UHDR and conventional (CONV) mode. Two phantoms were developed to irradiate *Drosophila melanogaster*. The characterization was based both on active (ultra-thin ion chamber prototype, scintillator) and passive detectors (radiochromic films, OSLD). Moreover, the UHDR capabilities for megavoltage photon were investigated with an additional dedicated phantom.

**Results:**

The electron UHDR irradiations provided average dose rates in the range of 200–––7500 Gy/s. The beam spatial uniformity within a single vial was better than ± 5 %. The dose delivered to *Drosophila* melanogaster in different configurations and beam modalities was confirmed to the ± 5 % level. The average dose rate achieved with photon megavoltage UHDR radiation reached beyond 40 Gy/s.

**Conclusions:**

This high-throughput experimental platform on a converted clinical linear accelerator could be used to compare CONV to UHDR for up to 500 animals per week for biological endpoints at up to 1000 Gy. The production of photon megavoltage UHDR radiation was also demonstrated for the first time at a converted clinical linac.

## Highlights

• Developed phantoms for the irradiation of Drosophila melanogaster.
• Converted a conventional linac to ultra-high dose-rate radiotherapy.
• The setup allowed comparison of conventional and Flash dose rates.
• Biological endpoints at up to 1000 Gy could be investigated.
• Production of photon megavoltage UHDR was demonstrated at a clinical linac.

## Graphical abstract

**Figure:**
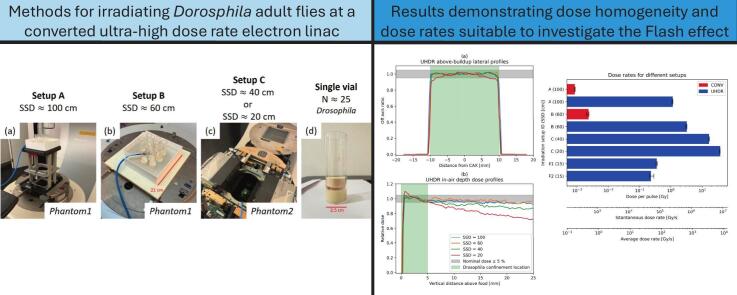

## 1 Introduction

The delivery of radiotherapy (RT) with ultra-high dose rate (UHDR, average dose rates ≳40 Gy/s) provides an opportunity to widen the therapeutic window [1,2]. This may be allowed by an increased sparing of normal tissue and unchanged tumour control experimentally observed with UHDR compared to conventional (CONV, average dose rates ≲30 Gy/min) RT, which is referred to as Flash effect [3]. The research field is rapidly evolving and the first in-human applications of electron [4] and proton [5] UHDR paved the way for further upcoming clinical trials focusing on the application of UHDR to patients with skin cancer [[6], [7], [8]] and bone [9,10] metastasis.

These clinical trials are backed up by multiple positive experiments demonstrating superior normal tissue sparing at UHDR in-vivo with several animal models including rodents [[11], [12], [13]], zebrafish [14], mini-pigs [15] and trials with domestic animals [16]. However, negative results were also reported for these animal [[17], [18], [19]]. The presence or absence of the Flash effect may provide insights on the underlying − yet to be deciphered − mechanism [[20], [21], [22]], in which the UHDR beam properties are reported to be relevant [11,23]. These can be investigated with dedicated experimental irradiation platforms providing a wide range of beam parameters [24,25]. The extension of these approaches to an even wider range of beam configurations can contribute to deciphering the Flash mechanism. Moreover, previous in-vivo results relied on animal models which should adhere to strict ethical regulation, limiting the scalability and throughput of the experiments [26,27]. Therefore, this study also focused on developing methodologies for applying these novel irradiation techniques to in-vivo experiments based on animal models with looser ethical requirements, allowing higher throughput.

The developments of the proposed UHDR irradiation platform were focused to be applicable to the fruit fly, *Drosophila melanogaster*, which has been extensively adopted for radiation research [[28], [29], [30]] but so-far has been investigated only scarcely [31] in the context of UHDR RT. Not only the relaxation of the ethical requirements is an advantage, but using flies as a model organism offers several technical benefits compared to vertebrate models. They are simple and cost-effective to maintain in laboratory environments, have a significantly shorter life cycle, produce abundant externally laid embryos, and can undergo various genetic modifications [32]. While a direct translation to clinical applicability is limited due to their anatomy, high required doses and too short lifecycle for late effects analysis; *Drosophila* are a promising and novel model to be investigated in the context of UHDR RT and can increase the throughput of the research investigating the Flash effect, which is required for widening the access to this experimental field [33].

To develop an irradiation platform, it is important to address that accessing biological endpoints with the fruit fly requires irradiations of high single fraction doses. A previously modified clinical linac could deliver up to a few Gray per pulse at isocenter [34], which converts to a maximum of < 150 Gy per UHDR beam-on session that is not sufficient for *Drosophila* experiments requiring thousands of Grays [[35], [36], [37], [38]]. Previous work at reduced Source to Surface Distance (SSD) showed that the dose could be increased by positioning the sample at the interface mount [24] or at the jaws level [39], reaching respectively up to 0.7 and 5 Gy/pulse, which was also insufficient for accessing biological endpoints with adult flies. These limitations were addressed in this work.

This study aimed to extend the capabilities and dose reporting methods of a previously UHDR-converted clinical electron linac to allow UHDR and CONV irradiation of *Drosophila* with the purpose of investigating the Flash effect.

## 2 Materials and methods

### 2.1 Ultra-high dose rate electron linac

The current study was performed with the linac FLEX extension for TrueBeam SN 1001 (Varian Medical Systems, a Siemens Healthineers company, Palo Alto, USA), which was a modified version of an electron linear accelerator that has been in clinical use until 2022. The detailed description of the conversion has been previously reported [34,40].

**Fig. 1:**
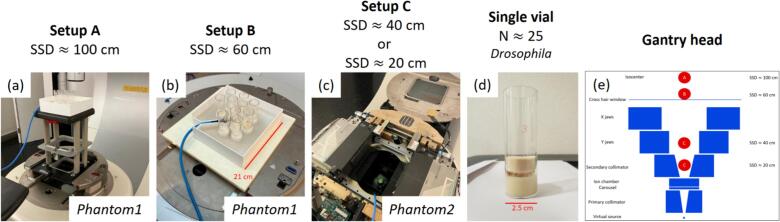
Overview of the setups investigated in the current study. (a) Setup A placed above the 20x20 cm^2^ electron cone; (b) Setup B placed at the interface mount; (c) Setup C placed at the jaws level and (d) single vial enclosing food with adult flies and a foam plug to contain the animals. The overview of the setups locations is also reported (e).

The results presented in the current study refer to the operation of the linac at 16 MeV CONV and UHDR. The current work reduced the SSD down to 20 cm aiming to reach beyond 1000 Gy per UHDR beam-on session, i.e. > 10 Gy/pulse. Fig. 1 shows the investigated setups. When reducing the SSD the jaw positions (XxY, in cm^2^ equivalent size at isocenter) were set respectively to: 18x18 for SSD = 100 cm, 40x40 for SSD = 60 cm, 26.8x29.2 for SSD = 40 cm and 40x36.2 for SSD = 20 cm.

### 2.2 Drosophila melanogaster preparation

This study investigated setups for the irradiation of adult *Drosophila*. The design of the phantoms aimed to irradiate flies of age 2–5 days after eclosion and the size up to 3 mm [41]. The preparation for irradiation included the following steps: polystyrene vials (0.1 cm thick walls) of cylindrical shape (2.5 cm diameter and 8.5 cm height) were chosen as transport devices and consecutively 2 cm of food (density 1.05 g/cm^3^) was located at the bottom of the vial. In this study, a detector was located above the food; or, up to 25 adult flies could be added to a single vial in potential future biological studies. The detector was locked in place by a foam plug (this study) or before the biology experiments, the animals were gently tapped down and constrained to a vertical space < 0.5 cm by placing a foam plug. The foam plug had density < 0.1 g/cm^3^ and contained the animals while allowing free flow of air, therefore delivering the RT at atmospheric oxygen levels.

### 2.3 Irradiation setups

Two phantoms were designed and could be set up at four different SSD (Fig. 1). *Phantom1* was 21x21 cm^2^, could host up to 10 vials simultaneously and could be positioned at isocenter (Setup A) or at the interface mount (Setup B). *Phantom2* was smaller such that could be position within the jaws (Setup C) and could host one vial at a time.

The objective of the design was manyfold: (i) maximising the number of vials irradiated per beam-on session for high throughput; (ii) minimising the dose variations due to differences in food preparation; (iii) utilising low-Z materials to limit sample activation; (iv) providing a simple but reproducible and robust approach to record the delivered dose and (v) delivering equivalent and homogeneous doses to all the animals receiving RT during the same beam-on session.

Objective (i) was achieved by a simultaneous irradiation of up to 10 vials and objective (ii) by ensuring sufficient buildup such that the *Drosophila* were close to the position of maximum dose deposition (R100) along the percentage depth dose (PDD). The 3D model was prepared in Fusion 360 (Autodesk, San Francisco, USA) and printed with a Kobra 2 Pro (Anycubiic, Hong Kong, China) using ecoPLA as material [42]. The phantoms were printed with 100 % infill leading to a nominal density of 1.24 g/cm^3^. The hollow structures were filled with Ecoflex GEL (SmoothOn, Macungie, USA) with density 1.06 g/cm^3^ (CT inspection). The choice of materials was determined by the design objective (iii) and further data regarding activation is provided in the supplementary paragraph A. The models are provided as open-source data for further use [43].

### 2.4 Detectors and calibrations

Several active and passive detectors were used in this study for the characterization of the irradiation setups. The phantom design objective (iv) was achieved by positioning an active detector in proximity of the irradiated vials (Fig. 1). The detector chosen for this purpose was a prototype of a ultra-thin parallel plates ion chamber (UTIC) (PTW, Freiburg, Germany) [44,45]. The charge collected by the UTIC was calibrated against passive detectors located inside the polystyrene vials, above 2 cm of Ecoflex GEL to mimic presence of food and without any backscattering to mimic the condition of the adult flies. The calibration factor *N* ([Gy/nC]) was corrected by *k_TP_* (variable day to day), but not by any other *k*_i_ (fixed for a given setup) [46]. The recombination correction was not applied since the UTIC is designed for a charge collection efficiency above 99 % with UHDR beams [44]. The phantom design objective (v) was verified by locating HD-V2 radiochromic films inside the vial at the *Drosophila* location, in air and without any back-scatter. The films were placed both along and orthogonal to the beam direction, matching the scenario of flies’ irradiation. The dose per pulse (DPP) was measured placing one at the time passive detectors (EBT3, HD-V2, OSLD) inside the vial at the *Drosophila* location and the readout by multiple independent detectors was used to estimate its value and related uncertainty. Moreover, the dose received for different vials-slots during simultaneous irradiations at SSD ≥ 60 cm was assessed with OSLD inserted in each vial.

The passive detectors were chosen among technologies that were proven to be applicable to UHDR beams: radiochromic films EBT3, HD-V2 [47,48] (Ashland, Bridgewater, USA) and the myOSLchip system (RadPro, Remscheid, Germany) [49]. The radiochromic films were evaluated after 24 h with transmission measurements using an Epson 12000XL flatbed scanner acquiring 48-bit RGB at 150 dpi, processed with FilmQA Pro 4.0 (Ashland, Bridgewater, USA) and the triple channel analysis. The calibration factor *N* was then verified by placing at the fly location the scintillator probe PRB-41 (SN355) read out by HyperScint RP-Flash (Medscint, Québec, Canada) [50]. The PRB-41 was chosen for this purpose because of its dimension (active detector diameter = 1 mm, length = 1 mm and distance from encapsulation surface = 2.5 mm) resembling the size of adult fruit flies. The passive detectors and the scintillator were calibrated in nationally recommended reference conditions [51] with 16 MeV electron CONV, which output was verified with a Roos chamber (SN1478, PTW, Freiburg, Germany) calibrated at the primary standard laboratory METAS. The uncertainty of the dose reporting was estimated to be 5 % for radiochromic films [52] and 3.5 % for OSLD [49]. Finally, the linearity of DPP of the active detectors was quantified against EBT3 films.

### 2.5 Exploratory endpoint: photon megavoltage UHDR irradiations

The production of photon megavoltage UHDR beams was also investigated as an explorative endpoint of this study. The dedicated *Phantom3* to position myOSLchip detectors down to SSD = 15 cm was designed and manufactured analogously to Phantom1-2. The phantom had two detector slots at depths 1 cm (P1) and 2 cm (P2) to allow for sufficient build-up. The linac bremsstrahlung target was extended to the position used by the modality 18X (5.8 cm) and 20 pulses of 16 MeV UHDR electron beam were delivered. We recorded the dose on the myOSLchip detectors to assess the maximum achievable DPP and dose rate for photon megavoltage UHDR beams. This detector was used for this explorative experiment with a geometry far from reference conditions based on previous studies reporting that BeO OSL have a negligible energy dependence for high-energy photon beams [53].

## 3 Results

### 3.1 Beam characteristics

**Fig. 2:**
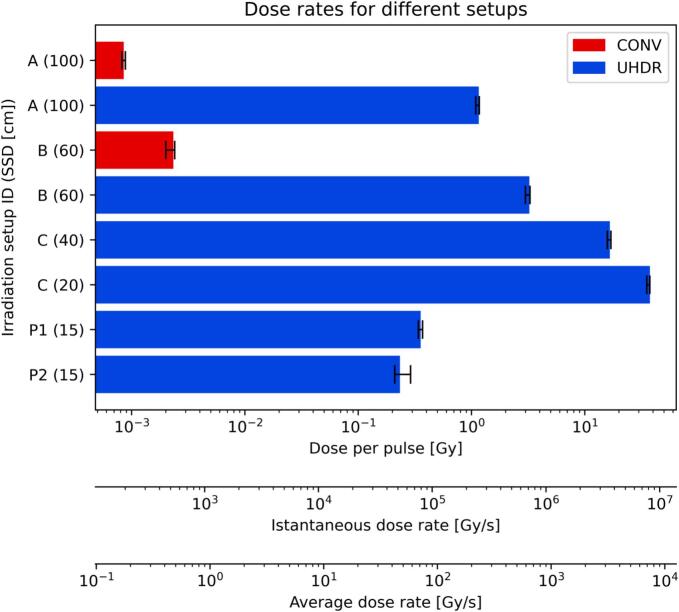
Breakdown of the DPP, average and instantaneous dose rates for different irradiation setups. For the setups for electron irradiations A-C the reported values correspond to the average among the passive detectors: EBT3, HD-V2 and myOSLchip. For the setups for photon irradiations P1-P2 the reported values correspond to the average of multiple repetitions of myOSLchip measurements. The error bars span the range minimum − maximum over the multiple measurements. Average and instantaneous dose rates were calculated based on the beam time-structure reported in Supplementary Table 2.

**Fig. 3:**
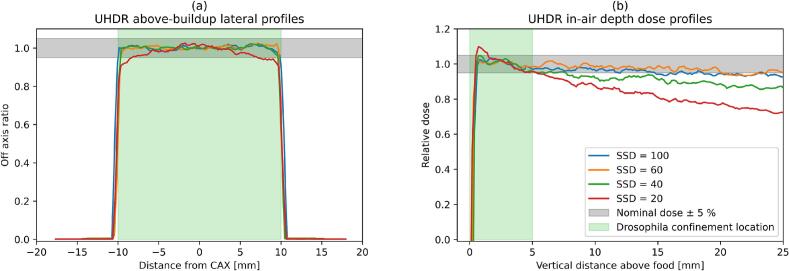
Dose profiles (a) orthogonal and (b) along beam direction profiles measured with HD-V2 films inside a vial at the location of the Drosophila without any back-scattering material. The measurements at SSD ≤ 40 cm were performed with the Phantom2, while a vial slot with 2.5 cm distance from the CAX within Phantom1 was used for SSD ≥ 60 cm, which were representative of all the vial locations used in given configurations. The green band indicates the region where the flies are confined during the irradiation. (For interpretation of the references to colour in this figure legend, the reader is referred to the web version of this article.)

The DPP, instantaneous and average dose rates reached up to 36.9 Gy, 8.2 ·10^6^ Gy/s and 7370 Gy/s, respectively, and the complete set of value is shown in Fig. 2 and Supplementary Table 1. Further beam parameters according to international recommendations [54] are reported in the Supplementary Table 2. For the setups at SSD ≥ 40 cm it could be observed in the UHDR beam profiles (Fig. 3) that at any potential location of the *Drosophila* during the beam-on the dose was uniform to the ± 5 % level. For the setup at SSD = 20 cm non-uniformity was observed both in the lateral and depth profiles.

### 3.2 Dose calibrations

**Fig. 4:**
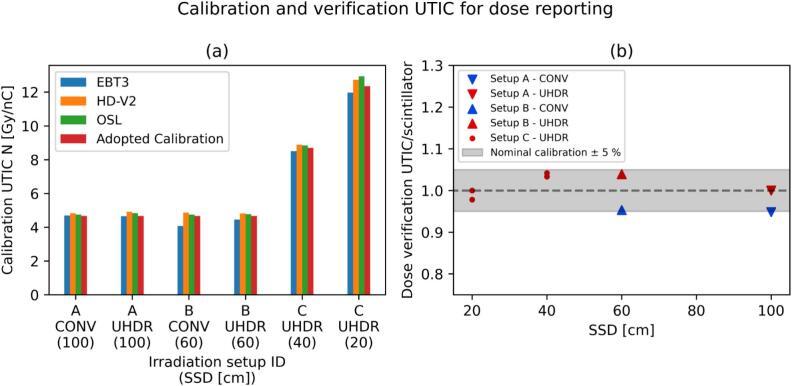
(a) Calibration values for the UTIC obtained against measurements performed with the passive detectors at the Drosophila melanogaster location. (b) Verification of the calibration N using the scintillator detector inside the polystyrene vials.

The calibration factors *N* for the UTIC to be used, after *k*_TP_ correction, for dose reporting to the *Drosophila* melanogaster were in the range 4.7–12.8 Gy/nC (Fig. 4a). We observed an agreement within ± 3 % over all setups for HD-V2 and the OSL and we adopted as calibration the average between these two detectors. The EBT3 led to deviations up to ± 6.5 %. The differences in *N* between UHDR and CONV for the setups A and B were + 1.8 % and −0.2 %, respectively. The differences between Phantom1 positioned at interface mount or on the electron cone were 0.7 %. The verification of the values for *N* with the scintillator confirmed the results within a ± 5 % uncertainty interval (Fig. 4b). The results assessing the applicability of the UTIC and the scintillator for UHDR radiation are reported in the Supplementary Fig. 1 and 2.

**Fig. 5:**
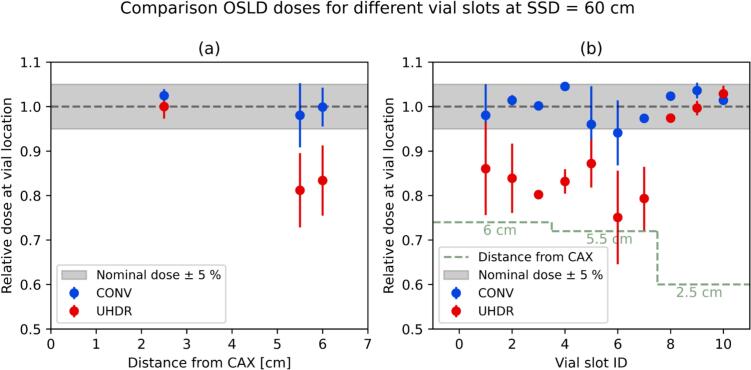
Doses recorded by myOSLchip detectors at different vial slots of Phantom1 during one CONV and one UHDR beam-on session. The doses (average and standard deviation) are reported with two different grouping. On the right (b) we report the individual measurement locations (3 detectors per slot) and on the left (a) grouped by the distance from the CAX (3–4 slots per point).

The results associated with the phantom design objective (v) for simultaneous irradiation of multiple vials showed an agreement within ± 5.5 % for all vial slots in CONV irradiations and an agreement within ± 2.5 % for the three vials with the closest distance from the central axes (CAX) for UHDR (Fig. 5). A drop of the dose down to −25 % was observed for UHDR at larger off-axis distances, which should therefore not be used for Drosophila irradiations.

### 3.3 Exploratory endpoint: Photon megavoltage UHDR irradiations

**Fig. 6:**
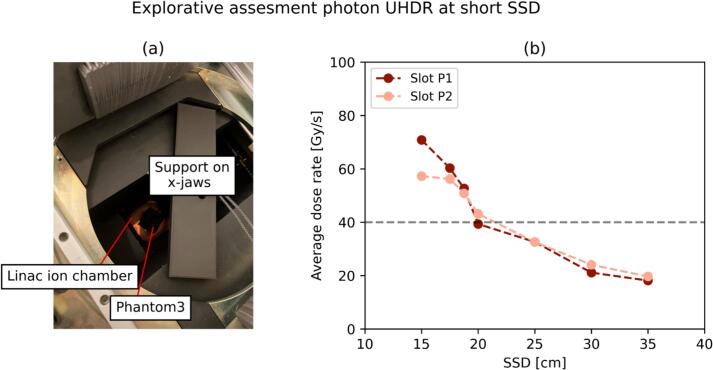
(a) Setup adopted for the investigation of the exploratory endpoint. (b) Average dose rate recorded by myOSLchip detectors at varying SSD.

The average dose rate achieved with photon megavoltage UHDR radiation reached beyond 40 Gy/s (Fig. 6). At the shortest SSD, DPP values up to 0.35 Gy/pulse were reached, which was confirmed by multiple (3) and independent myOSLchip detector irradiations. The most significant difference between the locations P1 and P2 was recorded at SSD = 15 cm, where the latter had a −19 % lower DDP.

## 4 Discussion

This study developed and commissioned a novel platform to investigate the Flash effect with *Drosophila melanogaster*, addressing dose, throughput, safety, and photon UHDR production challenges. *Drosophila melanogaster* is a highly versatile model for in-vivo experiments but it presents a significant higher radioresistance compared to vertebrates [[35], [36], [37], [38]]. The design of the experimental platform required therefore to achieve >1000 Gy in a single UHDR beam-on session while limiting the number of pulses to <99 to guarantee safety. Previous experimental platforms reported up to 5 Gy/pulse [24,39], which was insufficient for this purpose. The developments presented in this study were demonstrated to deliver up to 36.9 Gy/pulse (Supplementary Table 1). This was achieved by maximising the linac output [34] and reducing the SSD (Fig. 1). However, the Setup C at SSD = 20 cm presented several challenges: the activation of the linac head at this position was significantly higher than in other locations (Supplementary Fig. 3); the charge per pulse recorded by the UTIC reached beyond the range that was verified for linearity (Supplementary Fig. 1) and the beam profiles inside the vial had uniformity < 95 % due to the reduced beam divergence (Fig. 3a) and source-distance effect (Fig. 3b). On the other hand, the Setup C at SSD = 40 cm allowed the delivery of 16.4 Gy/pulse (up to 1623 Gy in 99 pulses) while solving all these concerns. Therefore, we conclude that the Setup C at SSD = 40 cm is the preferred configuration for UHDR irradiations of *Drosophila* melanogaster if > 1000 Gy are required for the biological endpoint. The design is shared in open-source version [43].

The generation of photon megavoltage UHDR radiation with dose rates above 40 Gy/s was demonstrated (Fig. 6). Previous photon UHDR experiments were reported at nuclear physics laboratories [55,56], in the kilovoltage range [57] and recently with a linac coupled with an optimized target [58]. The Phantom3 was optimised for this exploratory endpoint, further improvements allowing an online monitoring of the delivered dose may be investigated for the irradiation of *Drosophila* larvae or eggs (integral doses up to 35 Gy) held in smaller vials. Adult flies may not be irradiated in such modality due to biological endpoints requiring integral doses currently not accessible in photon UHDR mode.

The setups presented in the current study were optimised for *Drosophila* adult flies irradiations. Nonetheless, potential modifications may be applied to investigate the Flash effect with previously adopted biological models. Zebrafish irradiations may be performed by replacing the solid food in the vial with water and locating the embryos in it. A similar approach could be adopted for cells, spheroids or organoids irradiations. In these cases, the calibration procedure has to be repeated. The irradiation of murines would require different setups, which were previously developed and characterised for irradiations at interface mount [24] and within the jaws [39] and therefore outside the scope of this work.

It should also be noted that the present work had several limitations. First, the dose reporting for future experiments relies on the use of the UTIC and depends on the calibration factor N presented in Fig. 4, which had an uncertainty of 5 % dominated by the use of radiochromic films for absolute dose measurements, in particular if EBT3 were used above the recommended range of 10 Gy [59]. Then, the throughput (supplementary paragraph B) was not limited by the biological setup nor by the linac output, but rather by the accelerator workload and radiation protection aspects. Therefore, despite the most challenging scenario with 16 MeV being presented in this work, it may be beneficial adopting lower electron energies such as 9 MeV that would reduce the concerns regarding linac head activation. Also, the highest DPP achieved was 36.9 Gy/pulse but it was also shown its limited applicability due to non-uniformity of the profile and challenges in the dose monitoring. Also, the integral doses reported in the current study are higher compared to the ones commonly employed for Flash investigations and may lead to significant oxygen depletion [60] providing results that should be interpreted cautiously in the translation to humans. Finally, the generation of photon megavoltage UHDR radiation was demonstrated only as an exploratory endpoint. The results show that megavoltage UHDR is achievable and may be applicable for pre-clinical experiments, therefore a complete characterization of the beam properties and uncertainties is of interest for future studies.

This work presented an experimental platform to investigate the Flash effect with *Drosophila melanogaster*. This high-throughput setup could be used to compare CONV to UHDR RT for biological endpoints at up to 1000 Gy. UHDR RT with average dose rates above 3000 Gy/s could be employed with robust dose monitoring and uniform dose to all the flies in a vial. The production of photon megavoltage UHDR radiation was also demonstrated at a converted clinical linac.

## Declaration of competing interest

The authors declare the following financial interests/personal relationships which may be considered as potential competing interests: Nothing to declare for the current study. A previous study allowing the technical conversion of the linac to UHDR was partially supported by Varian Medical Systems.

## References

[1] Vozenin M.-C., Bourhis J., Durante M. (2022). Towards clinical translation of FLASH radiotherapy. Nat Rev Clin Oncol.

[2] Vozenin M.-C., Hendry J.H., Limoli C.L. (2019). Biological benefits of ultra-high dose rate FLASH radiotherapy: sleeping beauty awoken. Clin Oncol.

[3] Favaudon V., Caplier L., Monceau V., Pouzoulet F., Sayarath M., Fouillade C. (2014). Ultrahigh dose-rate FLASH irradiation increases the differential response between normal and tumor tissue in mice. Sci Transl Med.

[4] Bourhis J., Sozzi W.J., Jorge P.G., Gaide O., Bailat C., Duclos F. (2019). Treatment of a first patient with FLASH-radiotherapy. Radiother Oncol.

[5] Mascia A.E., Daugherty E.C., Zhang Y., Lee E., Xiao Z., Sertorio M. (2022). Proton FLASH radiotherapy for the treatment of symptomatic bone metastases: the FAST-01 nonrandomized trial. JAMA Oncol.

[6] Study Details | Irradiation of Melanoma in a Pulse | ClinicalTrials.gov n.d. https://clinicaltrials.gov/study/NCT04986696 (accessed June 26, 2024).

[7] Study Details | Randomized Phase II Selection Trial of FLASH Versus Conventional Radiotherapy for Patients with Localized Cutaneous Squamous Cell Carcinoma or Basal Cell Carcinoma | ClinicalTrials.gov 2025. https://clinicaltrials.gov/study/NCT05724875 (accessed June 30, 2025). 10.1016/j.ctro.2024.100743 PMC10867306 38362466

[8] von der Grün J. Study Details | A Phase I Clinical Study on Feasibility and Toxicity of LINAC-based Flash Radiotherapy for Palliative Treatment of Skin Lesions of Malignant Melanomas | ClinicalTrials.gov 2025. https://clinicaltrials.gov/study/NCT06549439 (accessed June 30, 2025).

[9] Study Details | Feasibility Study of FLASH Radiotherapy for the Treatment of Symptomatic Bone Metastases | ClinicalTrials.gov 2024. https://clinicaltrials.gov/study/NCT04592887 (accessed June 30, 2025).

[10] Study Details | FLASH Radiotherapy for the Treatment of Symptomatic Bone Metastases in the Thorax | ClinicalTrials.gov n.d. https://clinicaltrials.gov/study/NCT05524064 (accessed June 26, 2024).

[11] Montay-Gruel P., Petersson K., Jaccard M., Boivin G., Germond J.-F., Petit B. (2017). Irradiation in a flash: Unique sparing of memory in mice after whole brain irradiation with dose rates above 100 Gy/s. Radiother Oncol.

[12] Levy K., Natarajan S., Wang J., Chow S., Eggold J.T., Loo P.E. (2020). Abdominal FLASH irradiation reduces radiation-induced gastrointestinal toxicity for the treatment of ovarian cancer in mice. Sci Rep.

[13] Singers Sørensen B., Krzysztof Sitarz M., Ankjærgaard C., Johansen J., Andersen C.E., Kanouta E. (2022). *In vivo* validation and tissue sparing factor for acute damage of pencil beam scanning proton FLASH. Radiother Oncol.

[14] Pawelke J., Brand M., Hans S., Hideghéty K., Karsch L., Lessmann E. (2021). Electron dose rate and oxygen depletion protect zebrafish embryos from radiation damage. Radiother Oncol.

[15] Vozenin M.-C., De Fornel P., Petersson K., Favaudon V., Jaccard M., Germond J.-F. (2019). The advantage of FLASH radiotherapy confirmed in mini-pig and cat-cancer patients. Clin Cancer Res.

[16] Konradsson E., Arendt M.L., Bastholm Jensen K., Børresen B., Hansen A.E., Bäck S. (2021). Establishment and initial experience of clinical FLASH radiotherapy in canine cancer patients. Front Oncol.

[17] Bley C.R., Wolf F., Gonc P., Petit B., Bo T.T., Vozenin M.-C. (2022). Dose- and volume-limiting late toxicity of FLASH radiotherapy in cats with squamous cell carcinoma of the nasal planum and in mini pigs. Clin Cancer Res.

[18] Venkatesulu B.P., Sharma A., Pollard-Larkin J.M., Sadagopan R., Symons J., Neri S. (2019). Ultra high dose rate (35 Gy/sec) radiation does not spare the normal tissue in cardiac and splenic models of lymphopenia and gastrointestinal syndrome. Sci Rep.

[19] Beyreuther E., Brand M., Hans S., Hideghéty K., Karsch L., Leßmann E. (2019). Feasibility of proton FLASH effect tested by zebrafish embryo irradiation. Radiother Oncol.

[20] Labarbe R., Hotoiu L., Barbier J., Favaudon V. (2020). A physicochemical model of reaction kinetics supports peroxyl radical recombination as the main determinant of the FLASH effect. Radiother Oncol.

[21] Jansen J., Beyreuther E., García-Calderón D., Karsch L., Knoll J., Pawelke J. (2022). Changes in Radical Levels as a Cause for the FLASH effect: Impact of beam structure parameters at ultra-high dose rates on oxygen depletion in water. Radiother Oncol.

[22] Jin J.-Y., Gu A., Wang W., Oleinick N.L., Machtay M., Spring Kong F.-M. (2020). Ultra-high dose rate effect on circulating immune cells: a potential mechanism for FLASH effect?. Radiother Oncol.

[23] Liu K., Waldrop T., Aguilar E., Mims N., Neill D., Delahoussaye A. (2025). Redefining FLASH radiation therapy: the impact of mean dose rate and dose per pulse in the gastrointestinal tract. Int J Radiat Oncol Biol Phys.

[24] Byrne K.E., Poirier Y., Xu J., Gerry A., Foley M.J., Jackson I.L. (2024). Technical note: a small animal irradiation platform for investigating the dependence of the FLASH effect on electron beam parameters. Med Phys.

[25] Metzkes-Ng J., Brack F.-E., Kroll F., Bernert C., Bock S., Bodenstein E. (2023). The DRESDEN PLATFORM is a research hub for ultra-high dose rate radiobiology. Sci Rep.

[26] Abbott A. (2010). Lab-animal battle reaches truce. Nature.

[27] Tolba R.H. (2024). Current regulations in the Animal Welfare Act and the significance for animal research. Orthopadie Heidelb Ger.

[28] Su T.T. (2019). What drosophila can teach Us about radiation biology of human cancers. Adv Exp Med Biol.

[29] Porrazzo A., Cassandri M., D’Alessandro A., Morciano P., Rota R., Marampon F. (2023). DNA repair in tumor radioresistance: insights from fruit flies genetics. Cell Oncol Dordr.

[30] Schweizer P.M., Spanne P., Di Michiel M., Jauch U., Blattmann H., Laissue J.A. (2000). Tissue lesions caused by microplanar beams of synchrotron-generated X-rays in Drosophila melanogaster. Int J Radiat Biol.

[31] Hart A., Dudzic J.P., Clarke J.W., Eby J., Perlman S.J., Bazalova-Carter M. (2024). High-throughput, low-cost FLASH: irradiation of Drosophila melanogaster with low-energy X-rays using time structures spanning conventional and ultrahigh dose rates. J Radiat Res (Tokyo).

[32] Jennings B.H. (2011). *Drosophila* – a versatile model in biology & medicine. Mater Today.

[33] Moreau M., Mao S., Ngwa U., Yasmin-Karim S., China D., Hooshangnejad H. (2024). Democratizing FLASH radiotherapy. Semin Radiat Oncol.

[34] Dal Bello R., von der Grün J., Fabiano S., Rudolf T., Saltybaeva N., Stark L.S. (2023). Enabling ultra-high dose rate electron beams at a clinical linear accelerator for isocentric treatments. Radiother Oncol.

[35] Miquel J., Bensch K.G., Philpott D.E., Atlan H. (1972). Natural aging and radiation-induced life shortening in *Drosophila melanogaster*. Mech Ageing Dev.

[36] Sacher G.A. (1963). Effects of X-rays on the survival of drosophila imagoes. Physiol Zool.

[37] Zhang Y., Zhang Y., Shen C., Hao S., Duan W., Liu L. (2023). Ionizing radiation alters functional neurotransmission in Drosophila larvae. Front Cell Neurosci.

[38] Paithankar J.G., Deeksha K., Patil R.K. (2017). Gamma radiation tolerance in different life stages of the fruit fly Drosophila melanogaster. Int J Radiat Biol.

[39] Schüler E., Trovati S., King G., Lartey F., Rafat M., Villegas M. (2017). Experimental platform for ultra-high dose rate FLASH irradiation of small animals using a clinical linear accelerator. Int J Radiat Oncol Biol Phys.

[40] Dal Bello R., Yukihara E.G., Hohmann E., Guckenberger M., Tanadini-Lang S. (2024). Evaluation and applicability of radiation detectors for quantitative assessment of radiation exposure in a 16-MeV electron UHDR linac. Radiat Meas.

[41] Klok C.J., Harrison J.F. (2009). Atmospheric hypoxia limits selection for large body size in insects. PLoS One.

[42] ecoPLA - Black 1,75 mm / 1000 g. 3DJake UK n.d. https://www.3djake.uk/3djake/ecopla-black (accessed June 26, 2024).

[43] riccardodalbelloUSZ. riccardodalbelloUSZ/PhantomsFlashDrosophila 2024. https://github.com/riccardodalbelloUSZ/PhantomsFlashDrosophila (accessed June 27, 2024).

[44] Gómez F., Gonzalez-Castaño D.M., Fernández N.G., Pardo-Montero J., Schüller A., Gasparini A. (2022). Development of an ultra-thin parallel plate ionization chamber for dosimetry in FLASH radiotherapy. Med Phys.

[45] Kranzer R., Schüller A., Gómez Rodríguez F., Weidner J., Paz-Martín J., Looe H.K. (2022). Charge collection efficiency, underlying recombination mechanisms, and the role of electrode distance of vented ionization chambers under ultra-high dose-per-pulse conditions. Phys Medica PM Int J Devoted Appl Phys Med Biol Off J Ital Assoc Biomed Phys AIFB.

[46] Agency I.A.E. (2000). Absorbed dose determination in external beam radiotherapy. Int Atomic Energy Agency.

[47] Ashraf M.R., Rahman M., Zhang R., Williams B.B., Gladstone D.J., Pogue B.W. (2020). Dosimetry for FLASH radiotherapy: a review of tools and the role of radioluminescence and Cherenkov emission. Front Phys.

[48] Siddique S., Ruda H.E., Chow J.C.L. (2023). FLASH radiotherapy and the use of radiation dosimeters. Cancers.

[49] Bossin L., Dal Bello R., Christensen J.B., Schischke S., Motta S., Togno M. (2024). Performance of a BeO-based dosimetry system for proton and electron beam dose measurements. Radiat Meas.

[50] Poirier Y., Xu J., Mossahebi S., Therriault-Proulx F., Sawant A. (2022). Technical note: characterization and practical applications of a novel plastic scintillator for online dosimetry for an ultrahigh dose rate (FLASH). Med Phys.

[51] Haerle H., Hasenbalg F., Hirschi L., James L., Kohler G., Kottler C. (2019). Reference dosimetry of high-energy therapy electron beams with ionisation chambers. Swiss Soc Radiobiol Med Phys.

[52] Jaccard M., Petersson K., Buchillier T., Germond J.-F., Durán M.T., Vozenin M.-C. (2017). High dose-per-pulse electron beam dosimetry: usability and dose-rate independence of EBT3 Gafchromic films. Med Phys.

[53] Ando M., Hayashi H., Goto S., Yamaguchi H., Shimizu M. (2024). Beam quality conversion factor of BeO-OSLD for high-energy photon beams. Radiat Meas.

[54] Böhlen T.T., Psoroulas S., Aylward J.D., Beddar S., Douralis A., Del-pon G. (2024). Recording and reporting of ultra-high dose rate “FLASH” delivery for preclinical and clinical settings. Radiother Oncol.

[55] Esplen N., Egoriti L., Planche T., Rädel S., Koay H.-W., Humphries B. (2024). Dosimetric characterization of a novel UHDR megavoltage X-ray source for FLASH radiobiological experiments. Sci Rep.

[56] Yang Y., Wang J., Gao F., Liu Z., Dai T., Zhang H. (2024). FLASH radiotherapy using high-energy X-rays: current status of PARTER platform in FLASH research. Radiother Oncol.

[57] Cecchi D.D., Therriault-Proulx F., Lambert-Girard S., Hart A., Macdonald A., Pfleger M. (2021). Characterization of an x-ray tube-based ultrahigh dose-rate system for in vitro irradiations. Med Phys.

[58] Taylor E.R.J.F., Tullis I.D.C., Vojnovic B., Petersson K. (2025). Megavoltage photon FLASH for preclinical experiments. Med Phys.

[59] Liu K., Jorge P.G., Tailor R., Moeckli R., Schüler E. (2023). Comprehensive evaluation and new recommendations in the use of Gafchromic EBT3 film. Med Phys.

[60] Jansen J., Knoll J., Beyreuther E., Pawelke J., Skuza R., Hanley R. (2021). Does FLASH deplete oxygen? Experimental evaluation for photons, protons, and carbon ions. Med Phys.

