# Supplementary material for: Development and characterization of phantoms to investigate the Flash effect with Drosophila melanogaster at an ultra-high dose rate radiotherapy linac
Source: Phys Imaging Radiat Oncol. 2025 Sep 15;36:100835. doi: 10.1016/j.phro.2025.100835 (PMC12481075; doi:10.1016/j.phro.2025.100835)
Supplement: Supplementary Data 1 [file mmc1.docx]

**Supplementary Material**


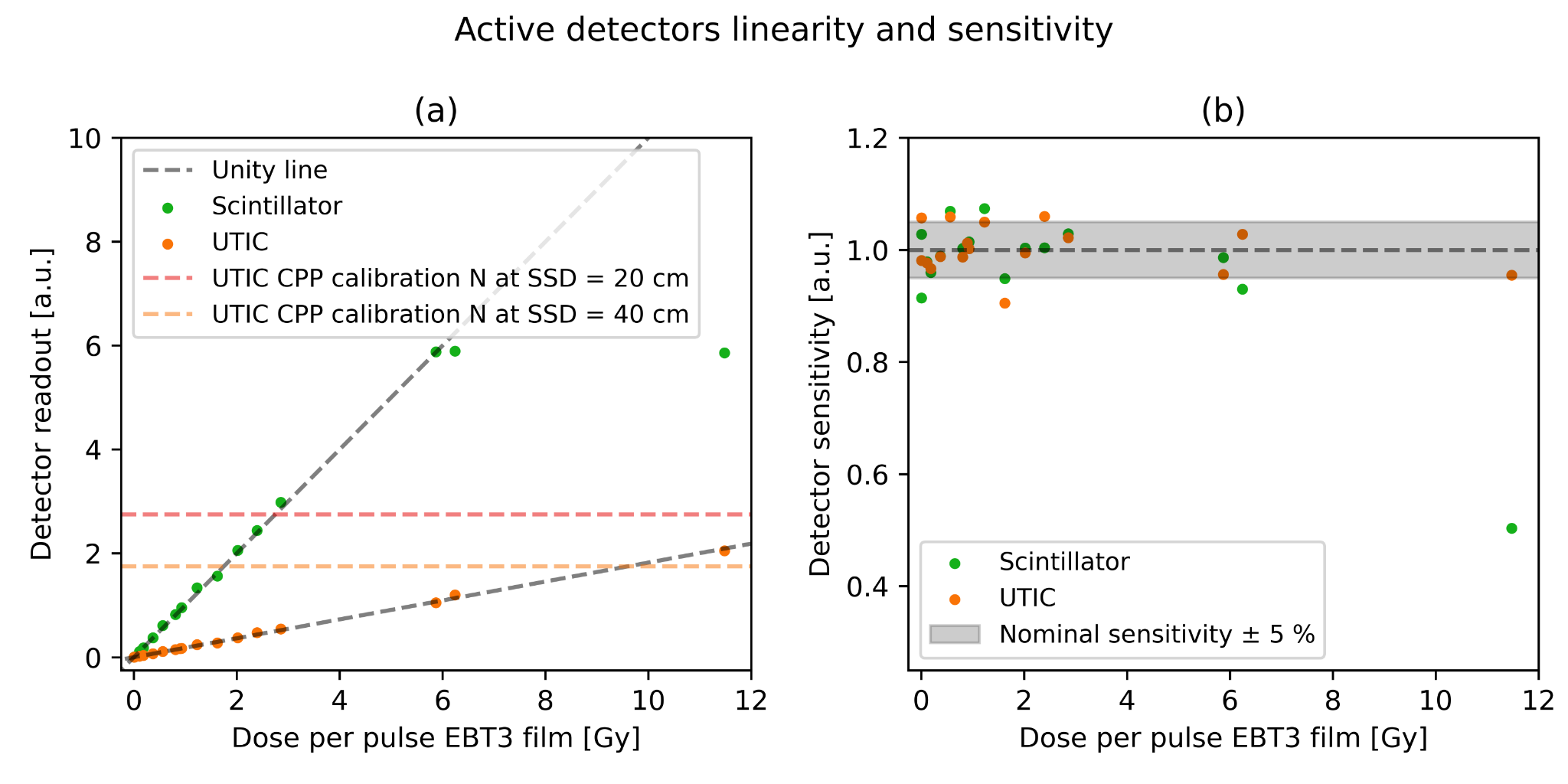


*Supplementary Figure 1. Linearity of response of the active detectors UTIC and scintillator against EBT3 films. We report the linearity (a) and the sensitivity (b). In (b) we also report in horizontal dashed lines the charge per pulse (CPP) recorded by the UTIC during the calibration of the Setup C at two different SSD.*


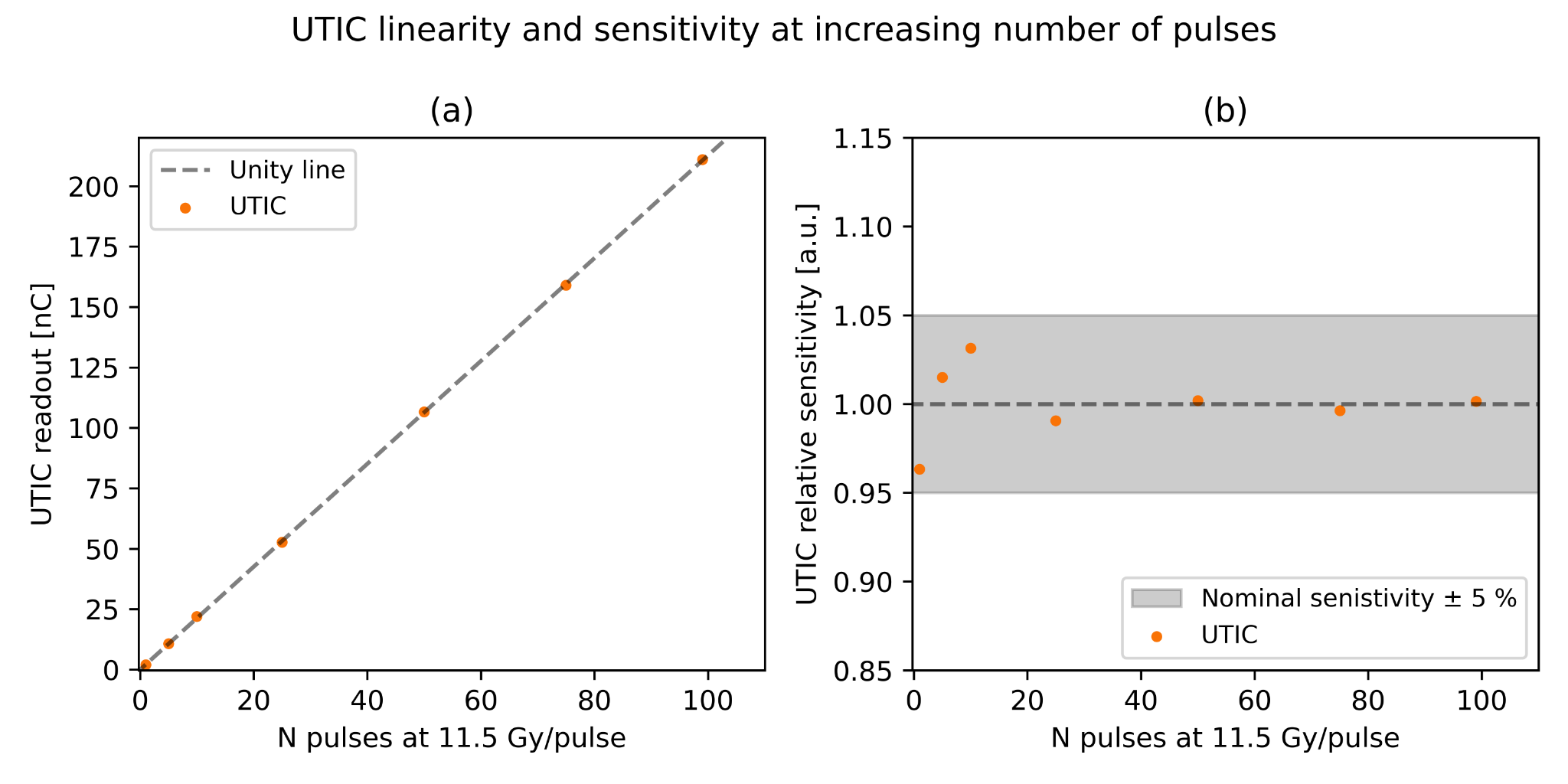


*Supplementary Figure 2. Linearity of response of the active detector UTIC for increasing number of pulses delivered at the highest achievable DPP for the detectors verification setup. We report the linearity (a) and the sensitivity (b).*


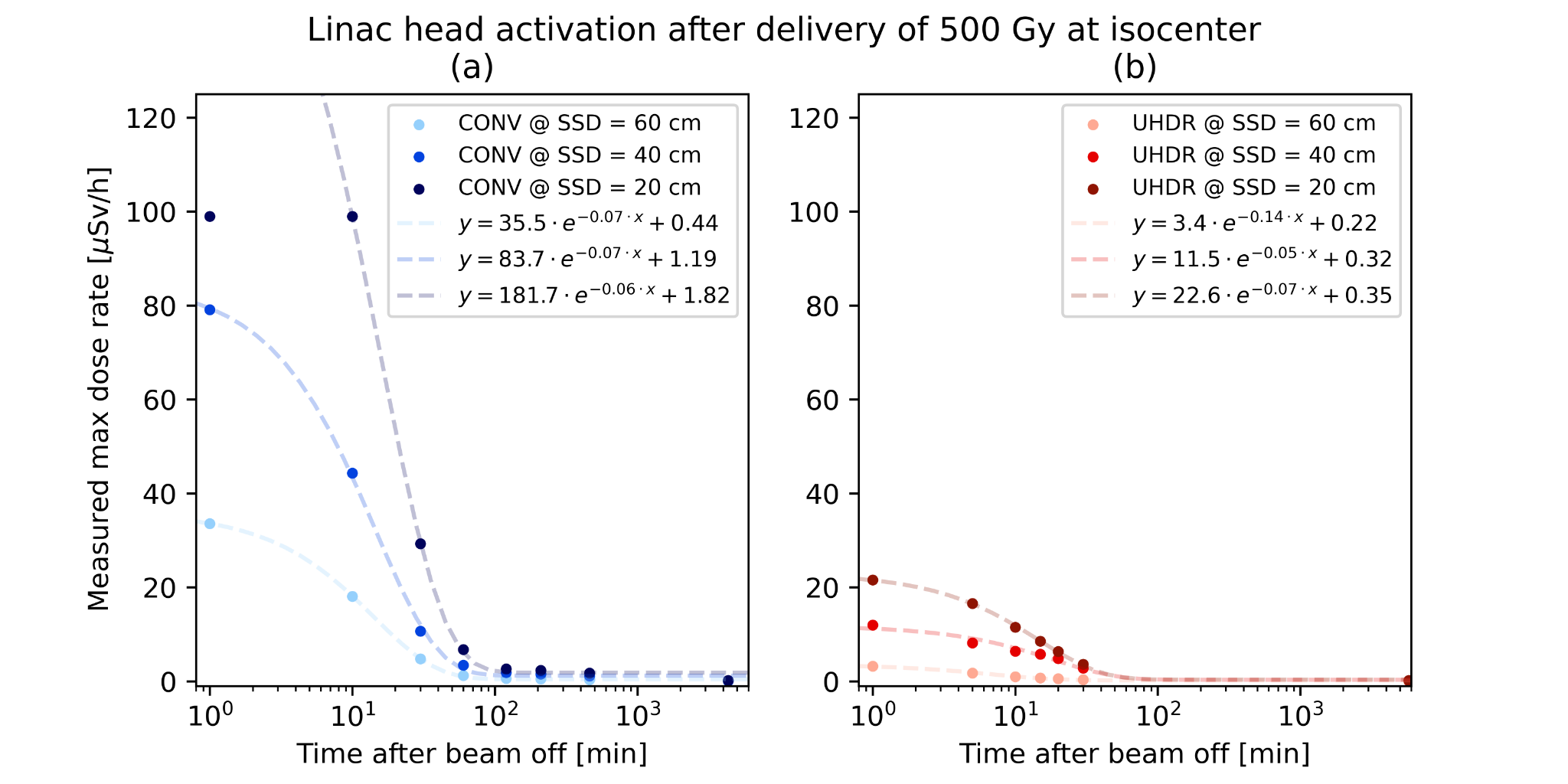


*Supplementary Figure 1. Activation of the linac head after irradiation of 50’000 monitor units in 16 MeV electron CONV mode (a) and 5 x 99 pulses in 16 MeV electron UHDR mode (b). Note on the fit (b) at SSD 60 cm: the activation in this setup is limited (x3.4 times less than at SSD 40 cm, x53 times less than CONV at SSD 20 cm) and comparable to the background (less than x10 the background), therefore the data uncertainty is higher and the fit quality lower, leading to a higher uncertainty in the determination of the time decay constant.*

| **Setup ID** | **SSD [cm]** | **Radiation quality** | **Linac modality** | **Dose per pulse [Gy]** | **Instantaneous dose rate [Gy/s]** | **Average dose rate [Gy/s]** |
| --- | --- | --- | --- | --- | --- | --- |
| A | 100 | 16 MeV electron | CONV | 8.4 ·10^-4^ (8.2·10^-4^ - 8.8·10^-4^) | 1.9 ·10^2^ (1.8·10^2^ - 2.0·10^2^) | 0.17 (0.16 - 0.18) |
| A | 100 | 16 MeV electron | UHDR | 1.14 (1.09 - 1.17) | 2.5 ·10^5^ (2.4·10^5^ - 2.6·10^5^) | 229 (219 - 233) |
| B | 60 | 16 MeV electron | CONV | 2.3 ·10^-3^ (2.0·10^-3^ - 2.4·10^-3^) | 5.0 ·10^2^ (4.4·10^2^ - 5.3·10^2^) | 0.45 (0.40 - 0.47) |
| B | 60 | 16 MeV electron | UHDR | 3.19 (2.99 - 3.28) | 7.1 ·10^5^ (6.6·10^5^ - 7.3·10^5^) | 638 (597 - 657) |
| C | 40 | 16 MeV electron | UHDR | 16.4 (15.8 - 17.0) | 3.7 ·10^6^ (3.5·10^6^ - 3.8·10^6^) | 3290 (3150 - 3410) |
| C | 20 | 16 MeV electron | UHDR | 36.9 (35-2 - 37.5) | 8.2 ·10^6^ (7.8·10^6^ - 8.3·10^6^) | 7370 (7050 - 7500) |
| P1 | 15 | 16 MV photons | UHDR | 0.35 (0.34 - 0.37) | 7.9 ·10^4^ (7.4·10^4^ - 8.3·10^4^) | 70.8 (67.0 - 74.6) |
| P2 | 15 | 16 MV photons | UHDR | 0.26 (0.21 - 0.29) | 5.8 ·10^4^ (4.6·10^4^ - 6.4·10^4^) | 51.9 (41.2 - 57.8) |

*Supplementary Table 1. Breakdown of the DPP, average and instantaneous dose rates for different irradiation setups. For the setups A-C the reported values correspond to the average (minimum - maximum) among the passive detectors: EBT3, HD-V2 and myOSLchip. For the setups P1-P2 the reported values correspond to the average (minimum - maximum) of multiple repetitions of myOSLchip measurements.*

| **Quantity** | **Abbreviation** | **Values** |
| --- | --- | --- |
| **Minimal reporting** | | |
| *General description* | | |
| Device name | FLEX estension for a TrueBeam v2.7.5 | |
| Accelerator type | Electron linac | |
| Dose delivery technique | Scattering and collimation | |
| Traceability and dosimetry code of practice used | Detectors cross calibrated at conventional dose rate to a PTW Roos chamber, which was calibrated in the Swiss primary standard laboratory METAS. Reference dosimetry performed according to SSRMP recommendation nr. 10. | |
| Additional key information about delivery | None | |
| Preclinical: Biological system(s), model(s), endpoint(s)  Clinical: Site, diagnosis, stage, cohort characteristics | Yellow-white Drosophila Melanogster. Multiple endpoints. | |
| Additional key information (including imaging) about irradiated systems/models/patients | The animals are contained in a cylindrical vial with food at the bottom and a cap at the top. The cap at the top allows air to flow freely but blocks the animals from escaping the vial. The animals live in an atmosphere that is equivalent to air. For the irradiation the animals are gently pushed down towards the food and the cap lowered such that they are confined in a space <5 mm along the beam direction (vertical dimension). In the plane orthogonal to the beam direction the animals are confined in a circular space of 20-25 mm diameter. Between 1h and 4h after the irradiation the animals are temporarily anaesthetised with CO2, the cap is lifted, and they are again free to move in a vertical space of > 2 cm. | |
| *Non-temporal beam parameters* | | |
| Radiation type and nominal beam energy | *E* | 16 MeV electrons |
| Beam dose at reference point or volume | *D*_beam_ | Multiple doses used. See main manuscript |
| Reference point or volume specification | *P*_ref_ or *V*_ref_ | Full uniform body dose to the Drosophila. Dose specified at the surface of the food, which corresponds to the location where the animals are confined during the irradiations. |
| Source-to-surface distance | SSD | Multiple SSD used. See main manuscript |
| Field size | FS | Multiple field sizes used. See main manuscript |
| *Temporal beam structure parameters* | | |
| Pause before next beam | *ΔT*_beam_ | At least 7.5 s |
| Beam-on time | *T*_beam_ | *#*_pulse_ x 5 ms |
| Number of pulses for beam | *#*_pulse_ | Multiple pulses number used. See main manuscript. |
| Pulse length^§^ | *t*_pulse_ | 4.5 µs |
| Pulse repetition frequency | *PRF* | 200 Hz |
| Pulse charge | *Q*_pulse_ | Approx 500 nC measured by inserting the photon bremsstrahlung target along the beam |
| Number of bunches per pulse | *#*_bunch_ | - |
| Bunch length | *t*_bunch_ | - |
| Bunch repetition frequency | *BRF* | - |
| Bunch charge | *Q*_bunch_ | - |
| **Optimal reporting** | | |
| *Derived and additional parameters* | | |
| Average dose rate at reference point | ADR | Multiple values. See Table 1 main manuscript  Calculated with: *D*_beam /_ *T*_beam_ |
| Instantaneous dose rate at reference point | IDR | Multiple values. See Table 1 main manuscript  Calculated with: *DPP / t*_pulse_ |
| Dose per pulse | DPP | Multiple values. See Table 1 main manuscript |
| Representative 2D dose distribution of beam, PDD and lateral profiles | - | Figure 3 |
| ADR-volume histograms of beam for relevant structures | - | - |

*Supplementary Table 2. Beam parameters according to the reporting recommendations.*

*Supplementary paragraph A. Radiation protection and activation*

Methods:

The activation of the linac head after delivery of RT to *Drosophila* was evaluated. We performed the measurements described hereinafter after the delivery of 50’000 monitor units (CONV) or 5 x 99 pulses (UHDR). This corresponded to approximately 500 Gy at the isocenter and 1500 Gy at the interface mount, which may be considered the upper limit for an experimental session. The detector Automess 6150AD-b/H (SN130605, Automess, Ladenburg, Germany) was positioned at the primary collimator location inside the gantry head (SSD = 20 cm), at the jaws level (SSD = 40 cm) and at the interface mount (SSD = 60 cm) and the *H**(10) was recorded.

Results:

The *H**(10) activities recorded after a beam-on delivering approximately 500 Gy to the isocenter are reported in Supplementary Figure 1. Fits with exponential functions were performed for the data points below the Automess 6150AD-b/H saturation point, i.e. 99 μSv/h. We observed up to a ten-times increase of the activation of the linac head after a CONV irradiation with respect to UHDR. The activity increased by a factor two or six when comparing it at the interface mount to SSD = 40 cm or 20 cm, respectively. Lower activations were observed for UHDR compared to CONV for the same isocenter dose, which is compatible with the number of electrons accelerated as previously reported^[[1]](#footnote-1)^.

Discussion:

Exposure of personnel should be carefully evaluated in the UHDR context. The workload of the presented experimental platform was limited to 1000 Gy/week to the isocenter, split between UHDR and CONV. A typical weekly experimental session investigating a biological endpoint requiring 1000 Gy may be performed as follows: 30’000 monitor units (approx. 300 Gy to isocenter) to be used for irradiating 10 vials simultaneously in CONV mode with Setup B; 600 pulses (approx. 600 Gy to isocenter) to be used for irradiating 10 vials consecutively in UHDR mode with Setup C and remaining 100 Gy to isocenter for quality assurance or calibrations. Scaling the data from Supplementary Figure 1 to these values and assuming a worse case scenario of immediate exposure of the personnel for 15 min^[[2]](#footnote-2)^ to the cumulative dose at SSD = 40 cm we obtain a cumulative dose of 20 μSv/week, which corresponds to the Swiss national limit of 0.02 mSv/week for the general public, and is below the allowed dose of 0.1 mSv/week to exposed personnel. These results show that *Drosophila* experiments are feasible and safe, but at the same time highlight the importance of evaluating personnel doses in the context of UHDR and high-dose experiments.

*Supplementary paragraph B. Experimental throughput.*

One of the advantages of experiments based on Drosophila is the high throughput of this biological model. To exploit it to its extent, this should be matched by elevated throughput during the irradiations, which was represented by the design aim (i) for this project. The UHDR RT with Setup C allowed to irradiate up to one vial (approx 25 flies) per beam-on session due to limitations in geometry at reduced SSD. Nonetheless, more than 1000 Gy could be delivered in sub-second intervals and exchanging vials for the following irradiation required less than one minute, which scaled up to 1’500 *Drosophila* irradiations per hour. Three vials could be simultaneously irradiated with UHDR for Setup B with equivalent doses (Figure 4) allowing up to 4’500 *Drosophila* to be irradiated per hour if the required biological endpoint could be achieved with less than 300 Gy. The CONV RT was more challenging. Setup A provided dose rates (10 Gy/min) that are impractical if integral doses of > 1000 Gy are required. Setup B should then be preferred. In this case, 10 vials (250 flies) could be simultaneously irradiated with equivalent doses (Figure 5) with 27 Gy/min, therefore requiring shortly more than half-hour for the delivery of 1000 Gy. This converts to a throughput of approximately 500 animals per hour. Therefore, if the biological endpoint requires < 300 Gy, the preferred option for irradiation of *Drosophila* was the Setup B for both CONV and UHDR. This was also supported by an equivalent calibration factor N for the UTIC for these two modalities (Figure 4a), which is justified by absence of saturation of the UTIC (Supplementary Figure 3) and matched beam qualities.

1. Dal Bello R, Yukihara EG, Hohmann E, Guckenberger M, Tanadini-Lang S. Evaluation and applicability of radiation detectors for quantitative assessment of radiation exposure in a 16-MeV electron UHDR linac. Radiat Meas 2024;176:107227. https://doi.org/10.1016/j.radmeas.2024.107227. [↑](#footnote-ref-1)
2. Ten times one minute for UHDR followed by one time five minutes for CONV [↑](#footnote-ref-2)
